# pH-Sensitive Folate-Targeted Liposomal Vitamin D Reprograms EZH2/STAT3 Axis and Induces Apoptosis, ROS, and Autophagy in MCF-7 Breast Cancer Cells

**DOI:** 10.34172/apb.46791

**Published:** 2026-07-01

**Authors:** Soheil Abbaspour-Ravasjani, Rahim Shahvalizadeh, Malahat Safavi, Mina Afrashteh Nour, Mahdi Talebi, Maryam Mohammadi, Hamed Hamishehkar

**Affiliations:** ^1^Student Research Committee, Department of Medical Nanotechnology, School of Medicine, Zanjan University of Medical Sciences, Zanjan, Iran; ^2^Drug Applied Research Center, Tabriz University of Medical Sciences, Tabriz, Iran; ^3^Department of Food Industry Science and Engineering, Faculty of Agriculture, University of Tabriz, Tabriz, Iran; ^4^Department of Biochemistry and Clinical Laboratories, Faculty of Medicine, Tabriz University of Medical Sciences, Tabriz, Iran; ^5^Student Research Committee, Tabriz University of Medical Sciences, Tabriz, Iran; ^6^Department of Applied Cell Sciences, Faculty of Advanced Medical Sciences, Tabriz University of Medical Sciences, Tabriz, Iran; ^7^Department of Food Science and Engineering, Faculty of Agriculture, University of Kurdistan, Sanandaj, Iran; ^8^New Material and Green Chemistry Research Center, Khazar University, 41 Mehseti Street, Baku, AZ1096, Azerbaijan

**Keywords:** pH-sensitive liposomes, Vitamin D3, Breast cancer, Folate targeting, MCF-7 cells

## Abstract

**Purpose:**

Vitamin D3 (Vit D3) possesses pleiotropic anticancer activity; however, its clinical application is limited by poor bioavailability and nonselective cellular uptake. This study aimed to develop folate (FA)-targeted, pH-sensitive liposomes for enhanced delivery of Vit D3 to breast cancer cells and to evaluate their effects on the VDR/EZH2/STAT3 signaling axis.

**Methods:**

Vit D3-loaded pH-sensitive liposomes were formulated and characterized for particle size, zeta potential, encapsulation efficiency, stability, and pH-responsive release. Cellular uptake, cytotoxicity, clonogenicity, apoptosis, reactive oxygen species (ROS) generation, autophagy, cell-cycle distribution, RT-qPCR, and Western blotting were evaluated in MCF-7 cells.

**Results:**

The optimized formulations exhibited nanoscale particle size, high encapsulation efficiency, physicochemical stability, and enhanced acidic pH-triggered release. FA-targeted liposomes significantly increased cellular uptake and cytotoxicity compared with free Vit D3 and non-targeted liposomes, with IC_50_ values of 15, 11, and 7.2 IU·mL^-1^, respectively. Vit D3-PEG/lipo-FA induced a 3.2-fold upregulation of VDR expression while suppressing EZH2 and STAT3 expression by 60% and 50%, respectively. In addition, treatment increased Caspase-3, LC3B, and NOX4 expression, promoted apoptosis and ROS generation, altered cell-cycle progression, and markedly reduced clonogenic survival. Western blot analysis confirmed these molecular alterations at the protein level.

**Conclusion:**

FA-targeted pH-sensitive liposomal delivery significantly enhances the anticancer efficacy of Vit D3 in MCF-7 cells by modulating the VDR/EZH2/STAT3 axis and activating apoptosis-, autophagy-, and ROS-associated pathways, highlighting its potential as a targeted nanotherapeutic strategy for breast cancer treatment.

## Introduction

 Breast cancer remains one of the most prevalent and clinically challenging malignancies worldwide, accounting for substantial morbidity and mortality among women despite advances in detection and multimodal therapy.^1^ Tumor heterogeneity, adaptive resistance mechanisms, and the complex interplay between oncogenic signaling and the tumor microenvironment continue to limit the efficacy of conventional chemotherapeutics and hormone therapies.^2^ Consequently, there is an ongoing need for therapeutic strategies that combine selective tumor targeting with molecularly informed interventions to suppress key drivers of proliferation and survival while minimizing systemic toxicity.^3^ Cholecalciferol (Vit D_3_) and its active metabolites have emerged as biologically pleiotropic regulators with potential anticancer activity.^4^ Beyond its classical role in calcium homeostasis, Vit D_3 _modulates cell proliferation, differentiation, apoptosis, and immune responses through the Vit D receptor (VDR), a nuclear transcription factor expressed in many tissues, including breast epithelium and breast cancer cells.^5^ Preclinical studies indicate that VDR activation can antagonize oncogenic signaling, induce cell-cycle arrest, promote programmed cell death, and modulate epigenetic regulators.^6^ Despite these promising activities, the clinical translation of Vit D_3_ as an anticancer agent has been limited by its lipophilicity, poor aqueous solubility, rapid systemic metabolism, and the need for high local concentrations to achieve robust antitumor effects in vitro.^7,8^ Delivering Vit D_3_ selectively to cancer cells and enhancing its intracellular bioavailability, therefore, represents an attractive strategy to potentiate its anticancer potential.^9,10^

 At the molecular level, two nodes of particular interest in breast cancer biology are EZH2 and STAT3. EZH2, the catalytic subunit of the Polycomb Repressive Complex 2 (PRC2), catalyzes trimethylation of histone H3 lysine 27 (H3K27me3) and acts as an epigenetic silencer of tumor suppressor genes; its overexpression is associated with aggressive phenotypes and poor prognosis in breast cancer.^11,12^ STAT3 is a central transcriptional mediator of cytokine and growth factor signaling that drives proliferation, survival, invasion, and immune evasion; persistent STAT3 activation is frequently observed in breast tumors and contributes to therapy resistance.^13,14^ Interactions between epigenetic regulators, such as EZH2, and signaling hubs, such as STAT3, create feed-forward circuits that reinforce oncogenic programs.^15^ Importantly, preclinical evidence suggests that modulation of VDR signaling can intersect with these pathways.^16^ For example, Vit D_3 _analogs have been reported to downregulate EZH2 expression and to inhibit STAT3 activation in some cancer models, highlighting the conceptual rationale for combining VDR activation with strategies that increase intracellular drug delivery.^17^ Nanoparticle-based drug delivery systems offer a solution to several of the pharmacological limitations of Vit D_3._^18^ Liposomes, in particular, are biocompatible vesicles capable of encapsulating hydrophobic agents within their lipid bilayer, improving solubility, protecting cargo from premature metabolism, and providing opportunities for surface functionalization to achieve active targeting.^19^ pH-sensitive liposomes are engineered to be relatively stable at physiological pH (7.4) but destabilize in the mildly acidic environments characteristic of tumors or endolysosomal compartments (pH ∼5.0–6.5), thereby promoting triggered release of the payload at the disease site or after endocytosis.^20^

 In this study, we developed folate-targeted, pH-sensitive liposomes to deliver Vit D_3_ selectively to MCF-7 breast cancer cells, addressing a gap where few studies jointly evaluate targeted, acid-triggered nanodelivery and downstream molecular effects. The study aimed to optimize Vit D_3_-loaded liposomes for folate-mediated uptake and pH-triggered release, and to test whether enhanced intracellular delivery strengthens VDR signaling while suppressing EZH2/STAT3 and inducing apoptosis, autophagy, ROS generation, and cell-cycle changes. Formulation characterization and a panel of cellular assays were used to link liposome properties with biological outcomes. The central hypothesis was that FA-targeted, pH-sensitive liposomes would deliver more Vit D_3_ into MCF-7 cells than free drug or non-targeted liposomes, resulting in stronger VDR induction, greater EZH2/STAT3 suppression, and lower clonogenic survival.

## Materials and Methods

###  Materials

 Methanol (≥ 99.9%, HPLC Grade), RPMI-1640-Medium, Tween 80^®^, Cholesteryl hemisuccinate (CHEMS), 4′,6-Diamidino-2-phenylindole dihydrochloride (DAPI), Anhydrous chloroform (≥ 99%), Isopropanol(≥ 99%), Vit D_3_, Penicillin-Streptomycin (Pen-Strep), Trypsin-EDTA solution (0.25%), 3-(4,5-Dimethyl-2-thiazolyl)-2,5-diphenyl-2H-tetrazolium Bromide (MTT), Fetal Bovine Serum (FBS), dialysis cellulose membrane bags (MWCO 12,000 Da), Monodansylcadaverine (≥ 99.0%), 2′,7′-Dichlorofluorescin diacetate (DCFH-DA), RNase A solution (20 mg.mL^-1^ in Tris buffer), Phosphate buffered saline tablets (PBS), TRI Reagent^®^, Triton X100, Dialysis tubing cellulose membrane (Mw = 14 KDa), Crystal violet solution (0.5%), Rhodamine B (≥ 95%), First Strand cDNA Synthesis Kit for RT-PCR (AMV), Propidium iodide (≥ 94.0%) and Annexin V-FITC/PI kit were all purchased from Sigma Aldrich Co. (Hamburg, Germany). 1,2-Dioleoyl-sn-glycero-3-PE (DOPE) and 1,2-distearoyl-sn-glycero-3-phosphoethanolamine-N-[folate(polyethylene glycol)-2000] (DSPE-PEG(2000) Folate) were obtained from Avanti Polar Lipids, Inc (Alabaster, AL).

###  Liposome Formulation and Preparation

 PEGylated folic-acid–targeted Vit D_3_ liposomes (Vit D_3_-PEG/lipo-FA) were prepared using the thin-film hydration technique.^20^ For untargeted PEGylated Vit D_3_ liposomes (Vit D_3_-PEG/lipo), DOPE, CHEMS, and DSPE-PEG2000 were combined at a molar ratio of 5.8:3.7:0.5 (total lipid mass = 10 mg). For the Vit D_3_-PEG/lipo-FA formulation, DOPE, CHEMS, DSPE-PEG2000, and DSPE-PEG2000-Fol were mixed at a molar ratio of 5.8:3.7:0.45:0.05 (total lipid mass = 10 mg). Lipids were dissolved in chloroform, and 1000 IU of Vit D_3 _and 1 mg of Tween 80^®^ were added to each formulation. For blank formulation (PEG/lipo), the DOPE, CHEMS, and DSPE-PEG2000 lipid combination was used without Vit D_3_. Finally, the solutions were transferred to a round-bottom flask, and the solvent was removed under reduced pressure to yield a dry lipid film. 100 µL of 0.1 M NaOH was applied to the film to ensure full ionization of CHEMS and to promote the formation of a lamellar structure, after which the film was hydrated with 300 mM ammonium sulfate at room temperature under vigorous stirring. The resulting liposomes were downsized by high-shear homogenization at 15,000 rpm and 65 °C for 20 minutes. External ammonium sulfate was removed using dialysis.

###  Physicochemical Characterization

 The mean liposome diameter was measured by dynamic light scattering (DLS) at 25 °C using a 90° scattering angle, and the zeta potential was measured by electrophoretic mobility under the same conditions. Size and zeta-potential determinations were performed in triplicate using a Nano ZS90 Zetasizer (Malvern Instruments, UK). Before analysis, samples were diluted tenfold with double-distilled, prefiltered water.

 The encapsulation efficiency (EE%) and drug loading (DL%) of Vit D_3 _were determined by HPLC (Knauer, Germany). Separation was carried out on a reversed-phase C18 column (250 × 4.6 mm, 5 µm) with UV detection at 265 nm, using a methanol: acetonitrile (20:80, v/v) mobile phase.

 To isolate the liposomal fraction for analysis, 100 µL of Vit D3-PEG/lipo-FA suspension was mixed with 100 µL of acetone to precipitate the liposomal material, then centrifuged at 12,000 rpm for 15 min. The resulting sediment was air-dried at room temperature, dissolved in chloroform, and then diluted tenfold with the mobile phase before HPLC analysis. Calibration was performed using four Vit D_3_ standard solutions (1–100 IU.mL^-1^) prepared in methanol: acetonitrile (80:20, v/v). The injection volume was 20 µL, and the mean retention time of Vit D_3_ was 7.51 min. Linearity parameters, including r^2^, slope, and intercept, were calculated. EE% and DL% were expressed as follows:


$$\begin{array}{l} EE\%=\frac{Total\;amount\;of\;encapsulated\;Vit\;D_{3}}{Total\;vitamin\;D_{3}\;added}\times 100\\ DL\%=\frac{Total\;amount\;of\;entrappedVit\;D_{3}}{Total\;mass\;of\;the\;drug-loaded\;liposomal\;formulation\;(lipid+entrapped\;drug)}\times 100 \end{array}$$

The morphology of the liposomes was examined by scanning electron microscopy (SEM; TESCAN; S8000; Czech Republic). Liposomal samples were diluted tenfold in double-distilled water, deposited onto aluminum foil, coated with gold, and imaged to visualize the surface structure.

###  Stability Studies

 The stability of Vit D_3_–PEG/lipo-FA was evaluated by monitoring changes in average particle diameter, polydispersity index (PDI), and drug retention during storage. The formulations were kept in sealed vials at refrigerated temperature (4–8 °C) for 60 days. At predetermined intervals of 0, 15, 30, 45, and 60 days, samples were collected and examined for particle size and PDI using dynamic light scattering. Drug retention was assessed by calculating the percentage of encapsulated Vit D_3_ remaining relative to the initial loading at each sampling time.^21^

###  In Vitro Release Study

*In vitro* drug release was evaluated by dialysis using cellulose membrane bags against HEPES–saline buffer containing 1% Tween 80^®^ at two pH values (5.5 and 7.4). Dialysis bags containing 500 µL of Vit D_3_–PEG/lipo-FA were immersed in 25 mL of the release medium and agitated at 37 °C. At 1, 2, 3, 4, 8, 12, 24, and 48 h, 500 µL aliquots of the external medium were collected and immediately replaced with an equal volume of fresh buffer to maintain sink conditions. The amount of Vit D3 released from the collected samples was quantified by HPLC, and cumulative release was calculated, accounting for sample removal and replacement.^22^

###  Hemocompatibility

 The hemocompatibility of Vit D_3_–PEG/lipo-FA was determined using a hemolysis test, which measures the release of hemoglobin from red blood cells (RBCs) following exposure to nanoliposomes. Human whole blood was collected in sterile EDTA tubes and centrifuged at 1,500 rpm for 5 min. The plasma and buffy coat were discarded, and the RBC fraction was washed and resuspended in phosphate-buffered saline (PBS). Liposomal samples were prepared in PBS at final concentrations of 0 (negative control), 0.5, 1, 2, 4, 6, 8, and 10 mg.mL^-1^ in a total volume of 1 mL. A 1% Triton X-100 solution was used as the positive control. The samples (Vit D_3_–PEG/lipo-FA plus RBCs) were incubated at 37 °C for 1 h and then centrifuged at 1,500 rpm for 5 min. The absorbance of the resulting supernatant was recorded at 450 nm, and the percentage of hemolysis was calculated using the following equation:


$$Hemolysed\;blood\left(\%\right)=\frac{Absorbance\left(sample\right)-Absorbance\left(PBS\right)}{Absorbance\left(Ctrl\;Pos\right)-Absorbance\left(PBS\right)}\times 100$$

###  Cell Culture and Treatment

 MCF-7 cells were obtained from the Pasteur Institute (Tehran, Iran) and cultured in RPMI-1640 medium supplemented with 10% FBS and 1% antibiotic solution. The cells were maintained at 37 °C in a humidified incubator with 5% CO₂. Subculturing was performed 24 and 48 h after seeding at an initial density of 4 × 10⁴ cells.mL^-1^. All experiments, including the MTT assay, flow cytometry, and fluorescence imaging, were initiated when the cultures reached approximately 70% confluence.

###  Cellular Uptake Assay

 The internalization of targeted liposomes was investigated in MCF-7 cells, which show high folate receptor expression, and A549 cells, which exhibit low folate expression, using flow cytometry (Becton Dickinson, USA). Cells were seeded in 12-well plates at a density of 4 × 10⁴ cells per well and allowed to attach overnight. Rhodamine B–labeled liposomal formulations (Rhodamine B-PEG/lipo and Rhodamine B-PEG/lipo-FA) were added at their respective IC₅₀ concentrations. At 0, 1, 2, 4, 8, 12, and 24 h after treatment, the medium was removed, the cells were trypsinized, washed twice with PBS, and analyzed by flow cytometry. Mean fluorescence intensity was recorded, and the results were processed with FlowJo software.^1^

###  Cytotoxicity Assay

 Cell viability and proliferation were assessed using the MTT assay to compare the effects of Vit D_3_, Vit D_3_-PEG/lipo, and Vit D3-PEG/lipo-FA. Cells were seeded in 96-well plates at 1.2 × 10⁴ cells per well and incubated overnight. To determine IC₅₀ values, the cells were exposed to serial dilutions of Vit D_3_, Vit D_3_-PEG/lipo, and Vit D_3_-PEG/lipo-FA for 48 h. After treatment, the medium was replaced with 100 µL of fresh medium and 50 µL of MTT solution (2 mg.mL^-1^ in PBS), followed by incubation for 4 h at 37 °C. The resulting formazan crystals were dissolved in 100 µL DMSO, and absorbance was measured at 570 nm using a microplate reader (Sunrise^TM^, Tecan, Switzerland).

###  Apoptosis Assays

 Apoptotic and necrotic cell populations were quantified using Annexin V/PI staining. MCF-7 cells were seeded in 6-well plates at a density of 4 × 10⁵ cells per well and incubated overnight. The cells were then treated with each formulation at its IC₅₀ concentration for 48 h. After treatment, the cells were collected by trypsinization, washed with PBS, and resuspended in 100 µL binding buffer. Staining with Annexin V/PI was carried out according to the manufacturer’s protocol, and the samples were subsequently analyzed by flow cytometry. In addition, apoptotic and necrotic cells were qualitatively examined under a fluorescence microscope after DAPI/PI staining (Olympus BX50).^23,24^

###  Colony Formation (Clonogenic) Assay

 A clonogenic assay was used to evaluate long-term antiproliferative effects. MCF-7 cells were plated at 2 × 10^3^ cells/well in 6-well plates and incubated for 48 h to allow attachment. Cells were then treated with EC_10_ concentrations of the formulations and maintained under standard culture conditions for two weeks. Colonies were fixed, stained with crystal violet, photographed, and counted using ImageJ software (NIH, Bethesda, MD).^25^

###  Cell Morphology Imaging

 To assess morphological changes induced by our treatments, brightfield imaging was performed. MCF-7 cells were seeded on 20 mm coverslips at a density of 2 × 10⁵ cells/well. After 48 h, cells were treated with formulations at their IC_50_ concentrations for an additional 48 h. Treated cells were washed twice with PBS, fixed in 4% formaldehyde for 4 h, washed again with PBS to remove residual fixative, and examined by brightfield microscopy.^26^

###  Cell Cycle Analysis

 Cell cycle distribution was analyzed in MCF-7 cells seeded in 6-well plates at 4 × 10⁵ cells per well. The cells were treated with Vit D_3_, Vit D_3_-PEG/lipo, and Vit D_3_-PEG/lipo-FA at their IC₅₀ concentrations for 48 h. Following trypsinization, the cells were fixed overnight in 70% ethanol. They were then treated overnight with 100 μg.mL^-1^ RNase A, 50 μg.mL^-1^ PI, and 0.1% Triton X-100, and finally analyzed by flow cytometry to determine DNA content and cell cycle phase distribution.^19^

###  Intracellular Reactive Oxygen Species Measurement

 Intracellular reactive oxygen species (ROS) production was determined using DCFH-DA staining followed by flow cytometry. MCF-7 cells were seeded in 6-well plates at 4 × 10⁵ cells per well and treated with the formulations at their 48-h IC₅₀ concentrations. After 48 h, the cells were incubated with 10 µM DCFH-DA prepared in serum-free RPMI for 30 min at 37 °C. The cells were then washed twice with PBS, detached, and analyzed by flow cytometry. Green fluorescence was detected in the FL1-H channel.^27^

###  Autophagy Assays

 Monodansylcadaverine (MDC) staining was employed to detect autophagic vacuoles and visualize autophagy activation in MCF-7 cells. Cells were seeded onto 12-mm coverslips at 2 × 10⁵ cells/well and incubated for 48 h. Cultures were then treated with formulations at their IC_50_ concentrations for 48 h, washed twice with PBS, and stained with 0.05 mmol. L⁻¹ MDC for 10 min at 37 °C. After three washes with PBS to remove excess dye, cells were examined by fluorescence microscopy.^23^

###  Gene Expression Analysis (RT-qPCR)

 Gene expression levels were measured by quantitative real-time PCR. MCF-7 cells were seeded at a density of 3 × 10⁴ cells per well and incubated overnight to allow attachment. The cells were then treated with the formulations at their 48-h IC₅₀ concentrations and incubated for an additional 48 h.

 Total RNA was extracted using TRI Reagent®. Briefly, 1.0 mL of TRI Reagent® was added to each well, and the lysate was allowed to stand for 20 min. The mixture was then centrifuged at 4 °C for 5 min, and the supernatant was transferred to a new tube. Next, 200 µL of chloroform was added, the sample was mixed gently, and incubated at −20 °C for 20 min. After centrifugation at 12,000 rpm for 15 min at 4–8 °C, the aqueous phase was recovered, and RNA was precipitated by adding 500 µL isopropanol followed by 10 min of incubation. The RNA pellet was washed with isopropanol, air-dried, and dissolved in 20 µL RNase-free water. RNA concentration and purity were determined using a NanoDrop 2000 spectrophotometer (Thermo Fisher Scientific).

 Complementary DNA (cDNA) was synthesized using the QuantiTect Reverse Transcription kit (Qiagen, Hilden, Germany). Quantitative PCR was performed with Power SYBR® Green PCR Master Mix (Applied Biosystems, Foster City, CA), using β-actin as the internal control. Relative expression of the target genes (VDR, EZH2, STAT3, CASP3, BCL2, LC3B, and NOX4) and β-actin was calculated by the 2−ΔΔCT method.^28^ Primer sequences are listed in Table 1.

**Table 1:** Primer sequences.

**Gene**	**Forward**	**Reverse**
β-actin	5'- CACCATTGGCAATGAGCGGTTC -3'	5'- AGGTCTTTGCGGATGTCCACGT -3'
VDR	5'-TCTCCAATCTGGATCTGAGTGAA-3'	5′-GGATGCTGTAACTGACCAGGT-3′
EZH2	5'- AGGACGGCTCCTCTAACCAT -3'	5'- CTTGGTGTTGCACTGTGCTT -3'
STAT3	5'- CAGCGAGAGCAGCAAAGAAG -3'	5'- GGAATGTCGGGGTAGAGGTAG -3'
CASP3	5′-GGAAGCGAATCAATGGACTCTGG-3′	5′-GCATCGAACATTGTACCAGACC-3′
BCL2	5'- ATCGCCCTGTGGATGACTGAGT -3'	5'- GCCAGGAGAAATCAAACAGAGGC -3'
LC3B	5'- CGGAGAAGACCTTCAAGCAG -3'	5'- CTGGGAGGCATAGACCATGT -3'
NOX4	5′-TAGATACCCACCCTCCC-3′	5′-GACTTATGACCGAAATGA-3′

###  Western Blotting Assay

 Western blot analysis was conducted to confirm the RT-PCR results. Following MCF-7 cells treatment with PEG/lipo, Vit D_3_, Vit D_3_-PEG/lipo, and Vit D_3_-PEG/lipo-FA, the cells were rinsed with ice-cold phosphate-buffered saline and lysed on ice for 30 min using a protein extraction buffer composed of 25 mM HEPES, 1% Triton X-100, 2 mM EDTA, 0.1 M NaCl, 25 mM NaF, 1 mM sodium orthovanadate, and a protease inhibitor cocktail (Roche). The lysates were then centrifuged at 12,000 × g for 20 min at 4 °C, and the resulting supernatants were collected. Protein concentrations were measured by the bicinchoninic acid (BCA) method. Equal amounts of protein from each sample were combined with loading buffer, denatured, separated by 12.5% SDS-PAGE, and transferred to PVDF membranes. To reduce nonspecific interactions, the membranes were blocked with 5% skim milk in Tris-buffered saline containing 0.1% Tween-20 (TBST), and then incubated overnight at 4 °C with the relevant primary antibodies. After washing, the membranes were exposed to horseradish peroxidase (HRP)-linked secondary antibodies for 1 h at room temperature. Protein bands were detected using an enhanced chemiluminescence (ECL) system (Bio-Rad). Band density was assessed by ImageJ software (version 1.54) and normalized against the corresponding loading control.^29^

###  Statistical Analysis

 All experiments were conducted in triplicate using independent biological replicates. Statistical processing was performed with GraphPad Prism software, version 10.6.1 (GraphPad Software, San Diego, CA). Depending on the experimental design, the data were analyzed using either an unpaired Student’s t-test or two-way analysis of variance (two-way ANOVA). When comparisons involved more than two groups, Tukey’s post hoc test was used to identify significant differences. A p value of less than 0.05 was considered to indicate statistical significance.

## Results

###  Liposome Formulation and Physicochemical Characterization

 All formulations displayed a narrow size distribution and negative surface charge. As shown in Figure 1, number-weighted mean diameters were 30.26 ± 10.14 nm for PEG/lipo, 61.28 ± 2.95 nm for Vit D_3_–PEG/lipo, and 83.54 ± 7.18 nm for Vit D_3_–PEG/lipo-FA. Corresponding zeta potentials were −16.3 ± 5.83 mV, −20.5 ± 7.66 mV, and −21.0 ± 7.10 mV, respectively. Electron microscopy (SEM) revealed predominantly spherical particles and supported the size measurements (Figure 1). Encapsulation efficiency (EE) of Vit D_3_ was very high for both drug-loaded formulations: 97.3 ± 3.06% for Vit D_3_–PEG/lipo and 98.2 ± 1.13% for Vit D_3_–PEG/lipo-FA. Drug loading (DL, sometimes reported as loading capacity, LC) values were 9.73 ± 0.30% and 9.82 ± 0.11% for Vit D_3_–PEG/lipo and Vit D_3_–PEG/lipo-FA, respectively.

**Figure 1:**
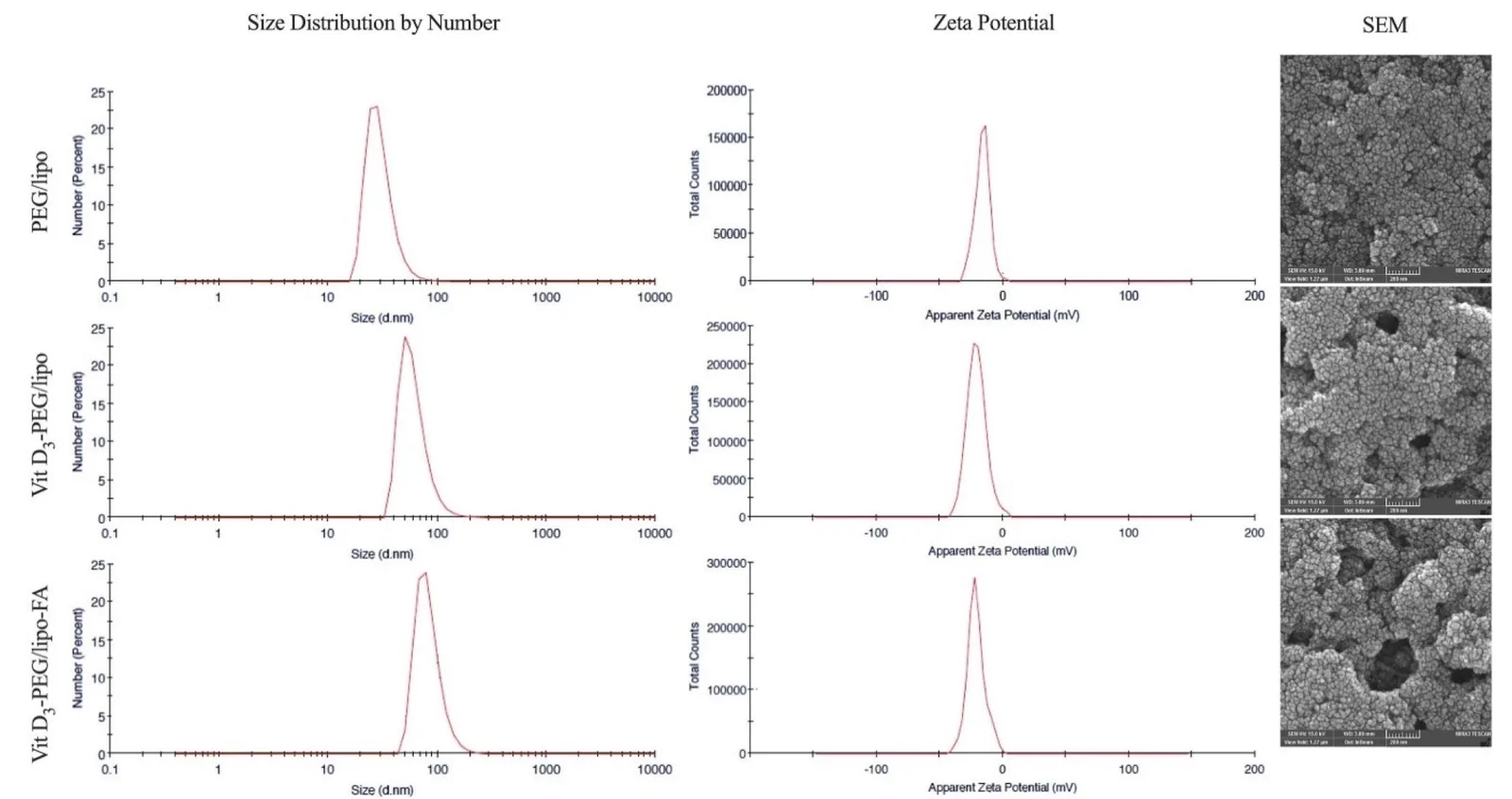
Physicochemical and morphological characterization of liposomal formulations. The left panels present the particle size distributions based on number for each formulation. The middle panels show the zeta potential distributions. The right panels display representative scanning electron microscopy (SEM) micrographs. The results indicate a uniform particle size distribution below 100 nm and a gradual change in surface properties after folic acid (FA) conjugation. Representative SEM images are shown, with scale bars indicated in each panel

###  Formulation Stability, in Vitro Release and Hemocompatibility

 Stability testing of Vit D_3_–PEG/lipo-FA over 60 days under refrigerated storage (4–8 °C) showed no substantial changes in mean hydrodynamic diameter or PDI across sampling points (Figure 2a). Leakage of Vit D_3 _was measured every 15 days (Figure 2b): cumulative release was 2.48 ± 0.52% at day 15, 4.24 ± 0.93% at day 30, 6.16 ± 1.31% at day 45, and 8.12 ± 1.12% at day 60. Statistical analysis indicated significant differences between day 0 and day 60, and between day 15 and day 60 (Figure 2b).

**Figure 2:**
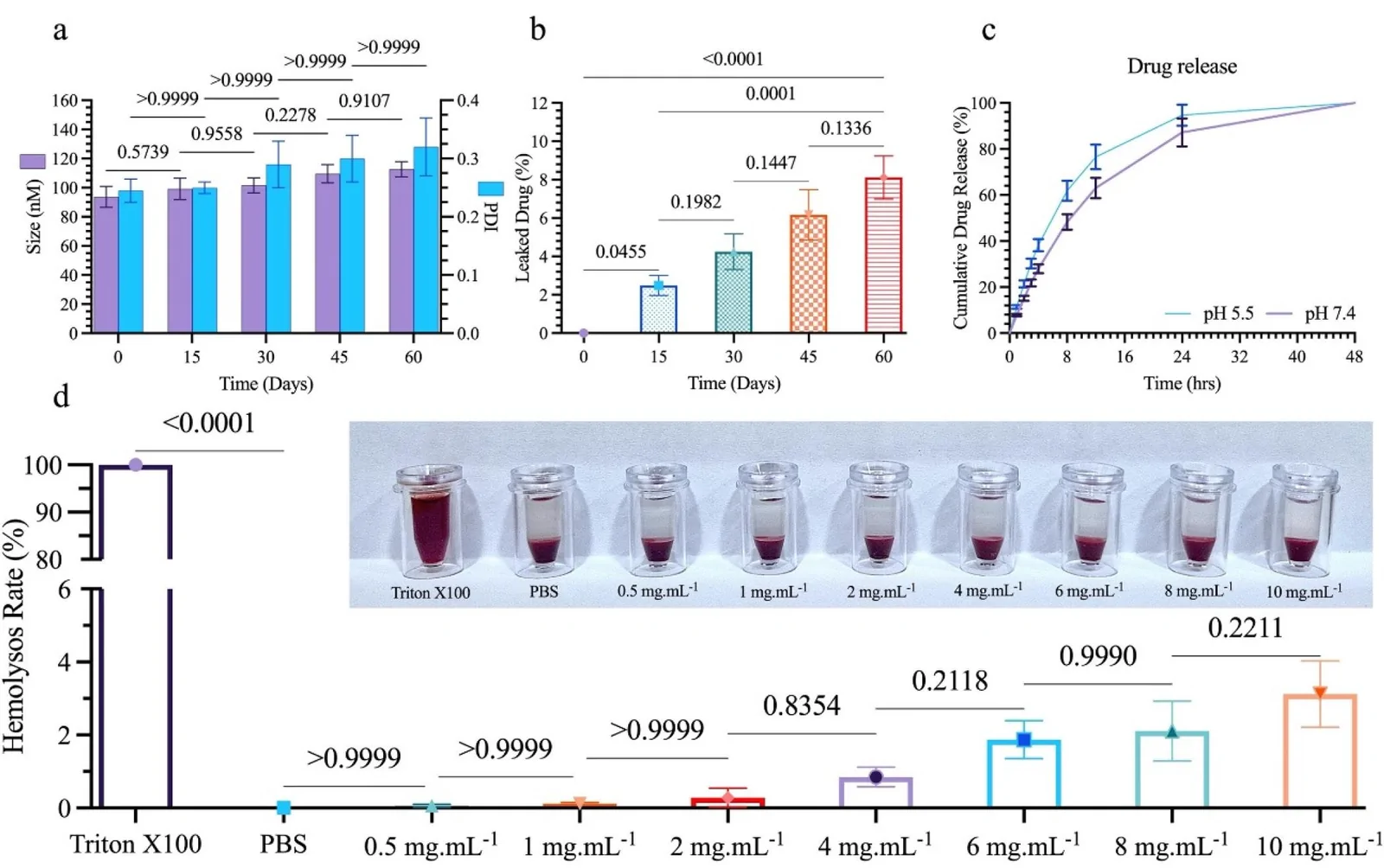
Stability, leakage, release and hemocompatibility. (a) Changes in hydrodynamic diameter and PDI of the Vit D3–PEG/lipo-FA during 60 days of refrigerated storage. (b) Cumulative leakage of Vit D_3_ from Vit D_3_–PEG/lipo-FA over 60 days. (c) Cumulative in vitro drug release profiles at pH 5.5 and pH 7.4. (d) Hemolysis (%) induced by the Vit D3–PEG/lipo-FA on human red blood cells. Data are reported as mean ± SD (n = 3). Statistical comparisons are indicated on the panels

 pH-dependent release of Vit D_3_–PEG/lipo-FA was assessed at pH 5.5 and pH 7.4 (Figure 2c). Release was similar at both pH values for the first hour, after which the formulation exhibited a time- and pH-dependent release profile. From 2 to 12 hours the drug release was significantly greater at pH 5.5 than at pH 7.4 (*P* < 0.05), consistent with destabilization of the nanosystem under acidic conditions and supporting potential selective release in acidic tumor microenvironments.

 Hemocompatibility testing (Figure 2d) showed minimal red-blood-cell hemolysis across the concentration range tested (0.5–10 mg.mL^-1^). Even at the highest concentration (10 mg.mL^-1^), PEG/lipo-FA induced only 3.12% hemolysis, which is negligible compared with the positive control (1% Triton X-100, defined as 100% hemolysis).

###  Cell Uptake

 Cellular internalization of Rhodamine-B–labeled non-targeted and FA-targeted liposomes was compared in MCF-7 (high folate receptor expression) and A549 (lower folate receptor expression) cells (Figure 3). Uptake by MCF-7 cells increased with time (Figure 3a), and FA-decorated liposomes showed markedly higher internalization than non-targeted liposomes (*P* < 0.0001). In A549 cells both targeted and non-targeted formulations were taken up (Figure 3b), but no statistically significant difference was observed between them (*P* > 0.05). These results indicate that folate decoration enhances uptake in folate-receptor–overexpressing cells, consistent with receptor-mediated endocytosis.

**Figure 3:**
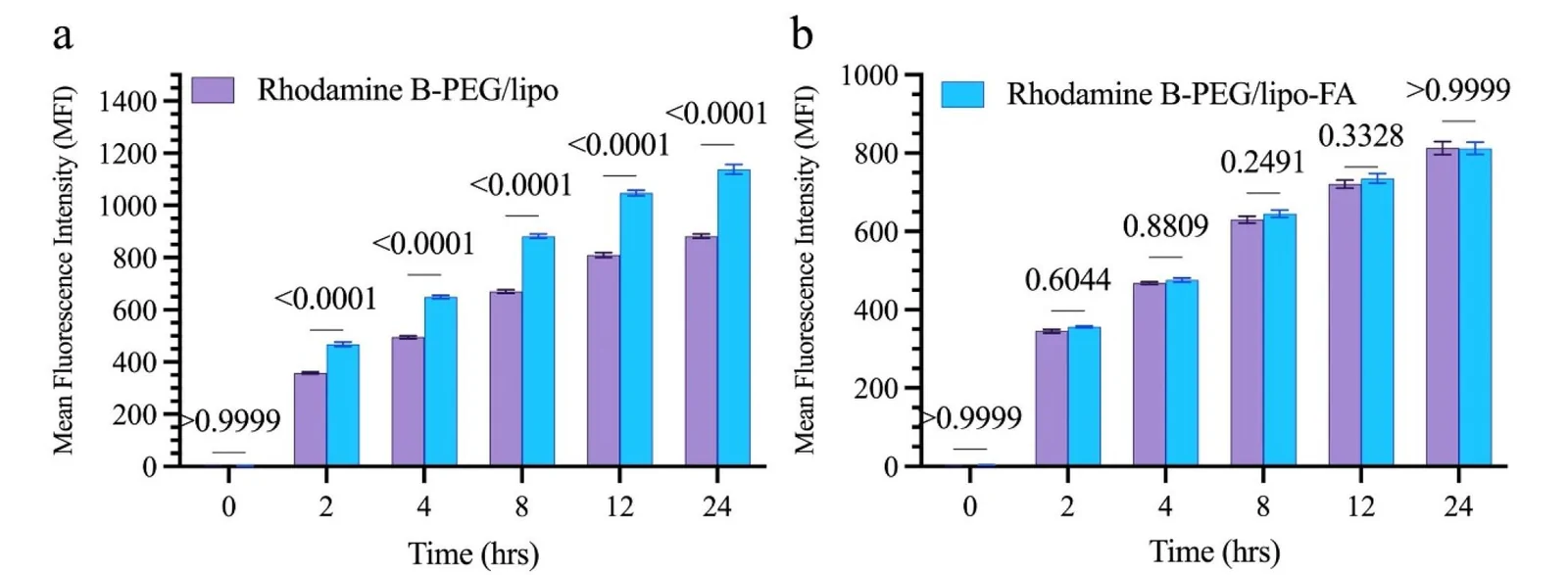
Cellular uptake measured by flow cytometry. Mean fluorescence intensity (MFI) of Rhodamine-B–labeled liposomes with and without FA decoration in MCF-7 and A549 cells at the indicated time points. Data are presented as mean ± SD (n = 3). Statistical comparisons are indicated on the panels

###  Cytotoxicity

 The cytotoxic effects after 48 h exposure are summarized in Figure 4. The MTT assay yielded IC₅₀ values of approximately 15 IU·mL⁻¹ for free Vit D_3_, ~11 IU·mL⁻¹ for the encapsulated (non-targeted) formulation, and ~7.2 IU·mL⁻¹ for the folate-targeted liposomal formulation. Encapsulation and subsequent folate targeting therefore produced progressive increases in cytotoxic potency against MCF-7 cells.

**Figure 4:**
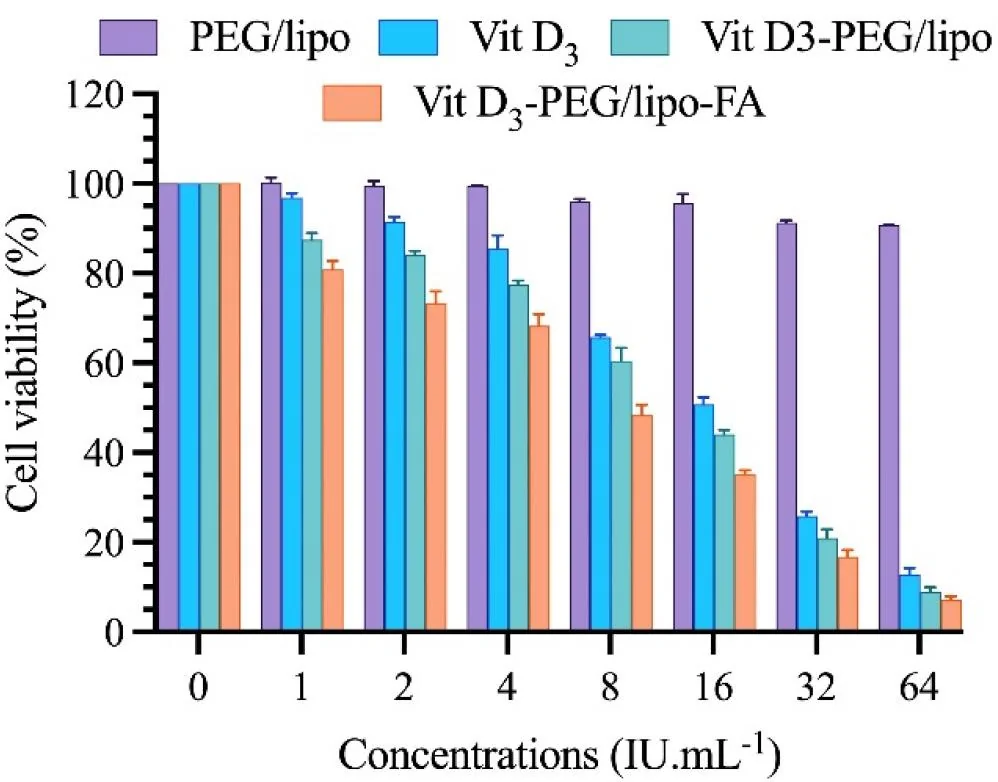
48-hour cytotoxicity. Dose–response effects of PEG/lipo, Vit D_3_, Vit D_3_–PEG/lipo and Vit D_3_–PEG/lipo-FA on MCF-7 cell viability (assessed by MTT) after 48 h. Data are presented as mean ± SD (n = 3)

###  Apoptosis, Necrosis and DNA Fragmentation

 Annexin V/PI analysis and nuclear staining were used to distinguish viable, apoptotic and necrotic populations (Figure 5). Treatment with free Vit D_3_ produced 14.9% necrosis, 2.38% early apoptosis and 22.4% late apoptosis. Encapsulation into liposomes significantly reduced necrosis to 3.05% (*P* = 0.0002), produced a nonsignificant increase in early apoptosis to 4.66% (*P* = 0.7922), and significantly increased late apoptosis to 30.9% (*P* = 0.0090) relative to free Vit D_3_. Addition of FA targeting produced a nonsignificant increase in necrosis (6.49%; *P* = 0.5292 versus Vit D_3_–PEG/lipo), a significant rise in early apoptosis to 22.2% (*P* = 0.0001 versus Vit D_3_–PEG/lipo), and a significant decrease in late apoptosis to 23.9% (*P* = 0.0395 versus Vit D_3_–PEG/lipo). DAPI/PI micrographs (Figure 5b) corroborated the flow-cytometry results by showing nuclear condensation and fragmentation consistent with apoptotic death.

**Figure 5:**
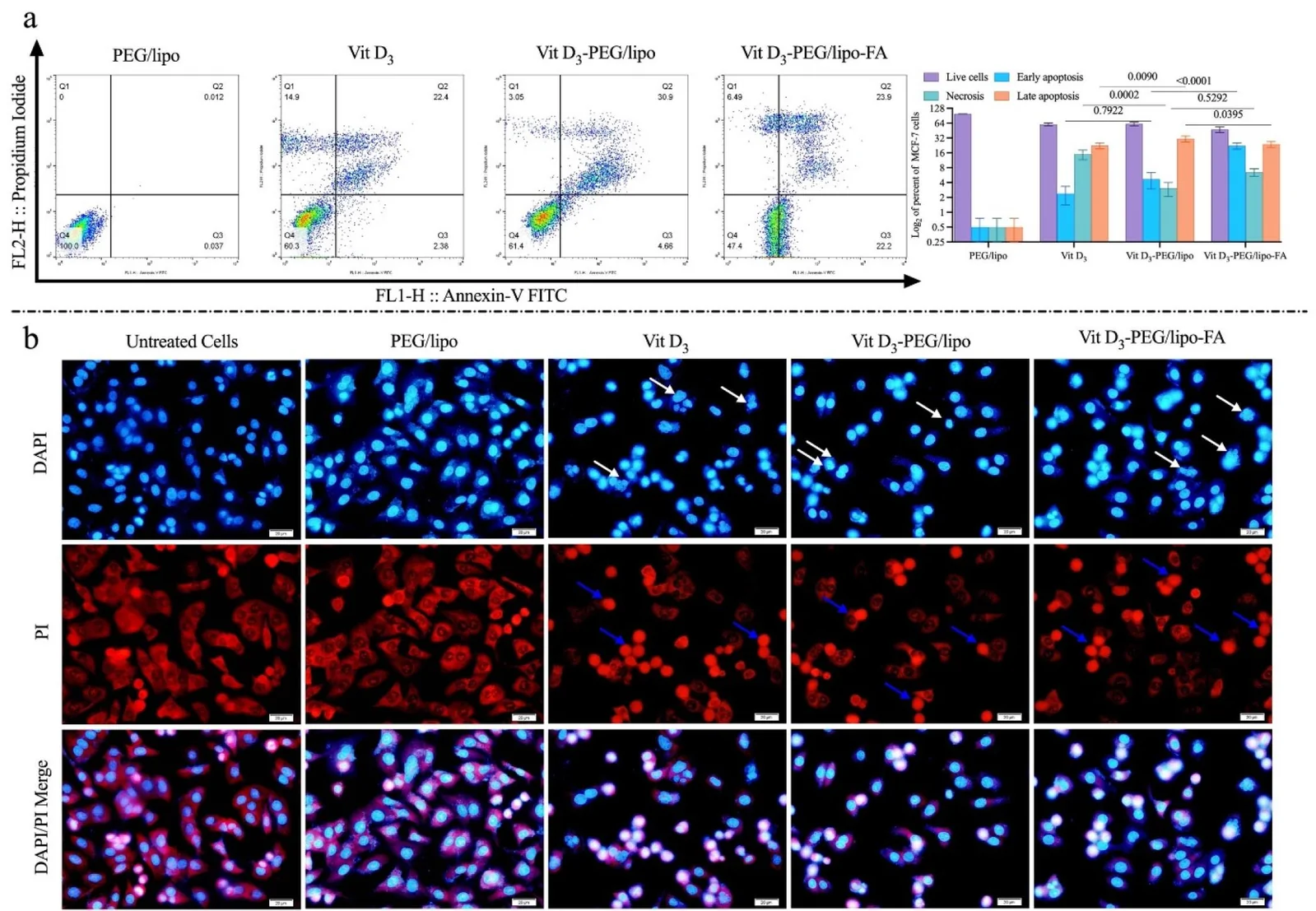
Apoptosis quantification and nuclear morphology. (a) Annexin V/PI flow-cytometric analysis showing percentages of viable, early apoptotic, late apoptotic and necrotic MCF-7 cells following treatment. (b) Representative DAPI/PI micrographs of treated cells (× 400 magnification) highlighting nuclear condensation and fragmentation. Quantitative apoptosis results are reported as mean ± SD (n = 3). Scale bars are shown in the images. White arrows indicate apoptotic nuclei; blue arrows indicate necrotic nuclei

###  Colony Formation, Cell Morphology and Autophagy Activation 

 Clonogenic assays assessed long-term antiproliferative effects (Figure 6a). Compared with the empty vehicle (PEG/lipo), free Vit D_3_ reduced colony number by approximately 54% (*P* < 0.0001). Encapsulation into liposomes produced an additional significant reduction in colony formation relative to free Vit D_3_ (*P* = 0.0014). FA targeting produced the strongest inhibition, yielding approximately 90% suppression of colony formation compared with the non-targeted formulation (*P* = 0.0010).

**Figure 6:**
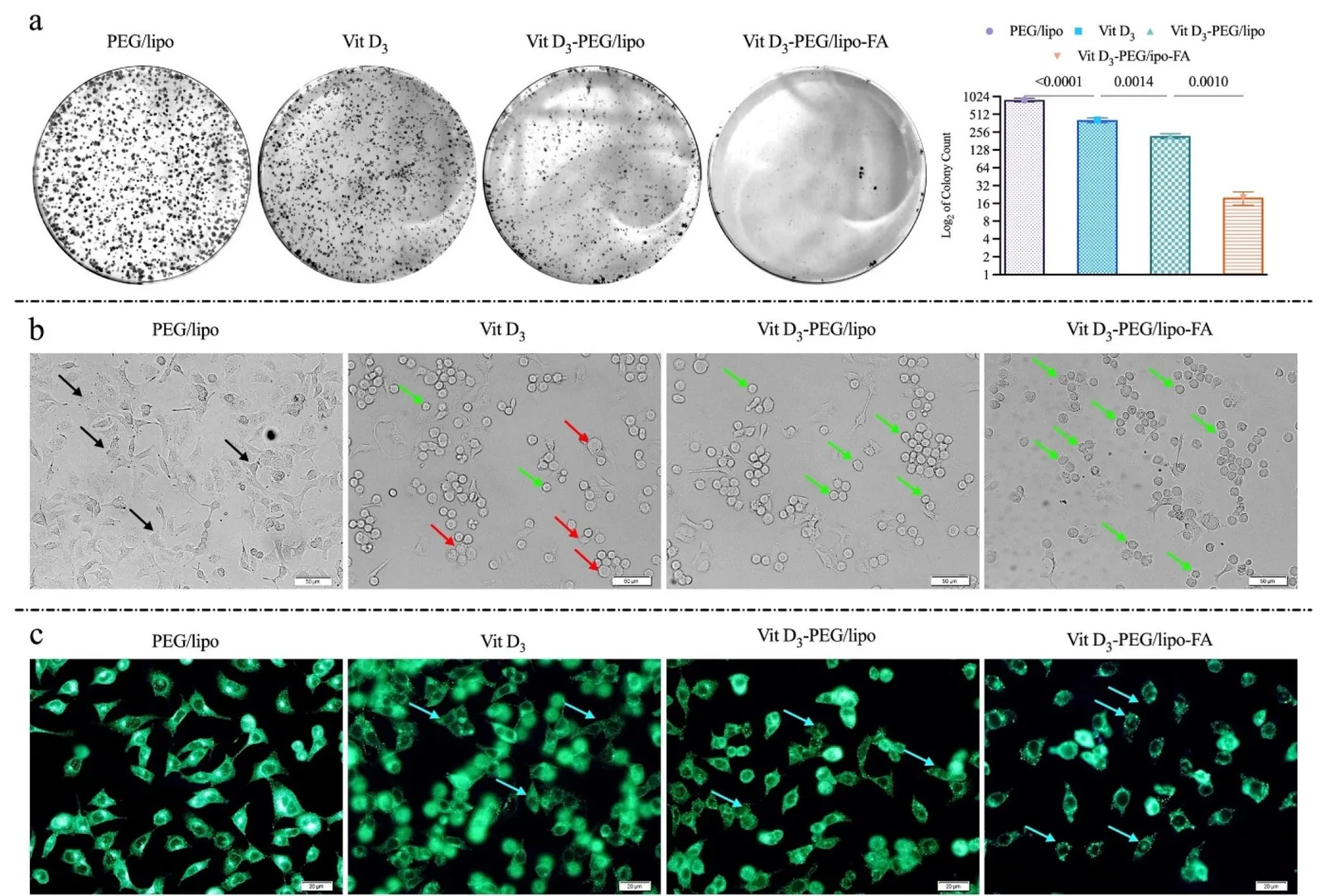
Long-term proliferation, morphology and autophagy. (a) Representative images and quantification of colony-formation assays showing the antiproliferative effect of each formulation after two weeks. (b) Brightfield images showing treatment-induced changes in MCF-7 cell morphology (200 × magnification). (c) Fluorescence micrographs showing MDC-stained autophagic vacuoles in MCF-7 cells (400 × magnification). Colony counts are reported as mean ± SD (n = 3). Scale bars are shown. Black arrows indicate morphologically normal cells; red arrows indicate necrotic cells; green arrows indicate apoptotic cells; turquoise (cyan) arrows indicate autophagic vacuoles

 Morphological analysis (Figure 6b) showed that cells treated with free Vit D_3_ exhibited both necrotic and apoptotic features. Encapsulation reduced the proportion of necrotic cells while increasing apoptotic morphology. Cells treated with Vit D_3_–PEG/lipo-FA displayed the greatest degree of cytoplasmic granulation and morphological alteration among groups.

 Autophagy activation was evaluated qualitatively by MDC staining (Figure 6c). Control cells (PEG/lipo) exhibited diffuse MDC fluorescence, whereas cells treated with Vit D_3_–PEG/lipo and Vit D_3_–PEG/lipo-FA showed a punctate MDC staining pattern indicative of autophagic vacuole formation. The FA-targeted formulation induced more pronounced puncta than non-targeted liposomes or free Vit D_3_, suggesting stronger induction of autophagy.

###  ROS Induction

 Intracellular ROS levels were measured by DCFH-DA staining and flow cytometry (Figure 7). Treatment with free Vit D_3_ significantly increased ROS levels by ~51 % relative to PEG/lipo controls (*P* = 0.0376). Encapsulation significantly augmented ROS production relative to free Vit D_3_ (~111% increase; *P* = 0.0001). FA targeting further and significantly increased ROS compared with the non-targeted liposomal formulation (~27% increase; *P* < 0.0003). These data indicate that encapsulation and folate targeting amplify Vit D_3_-induced oxidative stress in MCF-7 cells.

**Figure 7:**
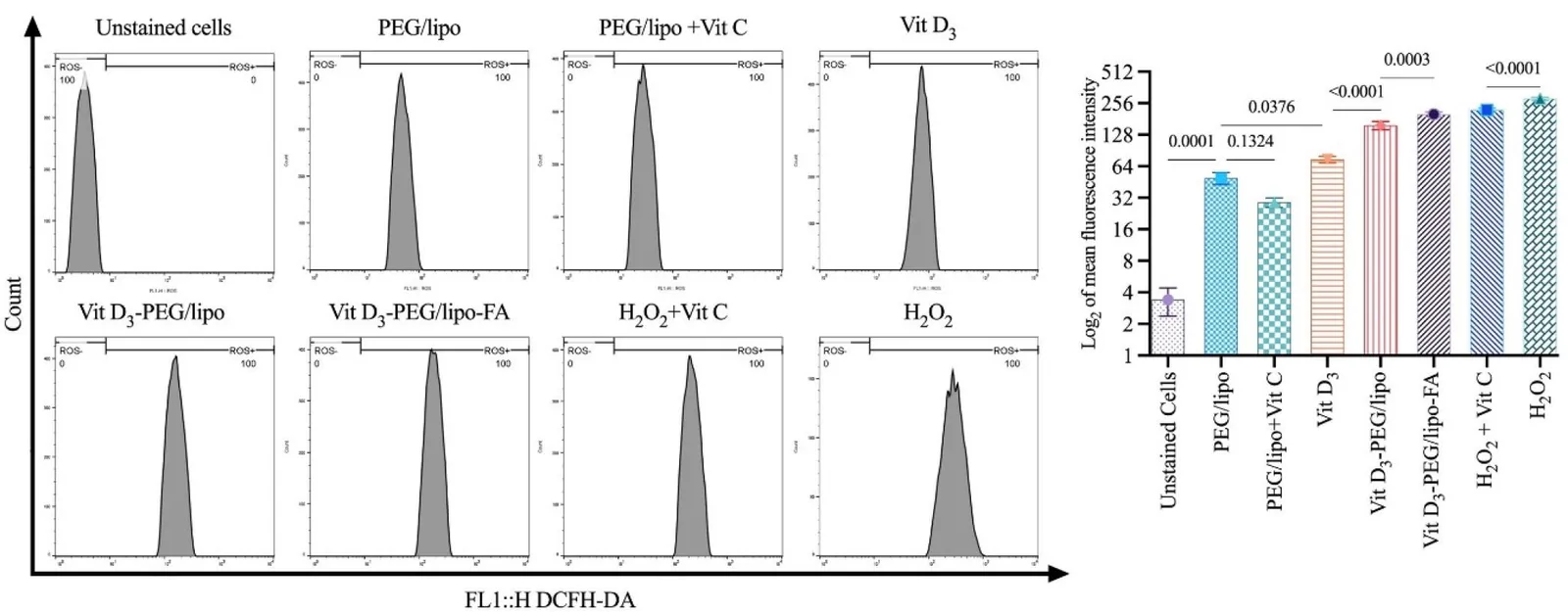
Oxidative stress induction. Flow-cytometric analysis of intracellular reactive oxygen species (ROS) in MCF-7 cells after treatment with the indicated formulations; results are expressed as mean fluorescence intensity (MFI). Data are presented as mean ± SD (n = 3)

###  Folate-Targeted Liposomal Vit D3 Alters Cell-Cycle Distribution and Modulates the VDR/EZH2/STAT3 Axis, Apoptosis, Autophagy, and Oxidative Stress

 Cell-cycle distribution and relative gene and protein expression were assessed after 48 h treatment with PEG/lipo, free Vit D_3_, Vit D_3_-PEG/lipo, and Vit D_3_-PEG/lipo-FA (Figure 8).

**Figure 8:**
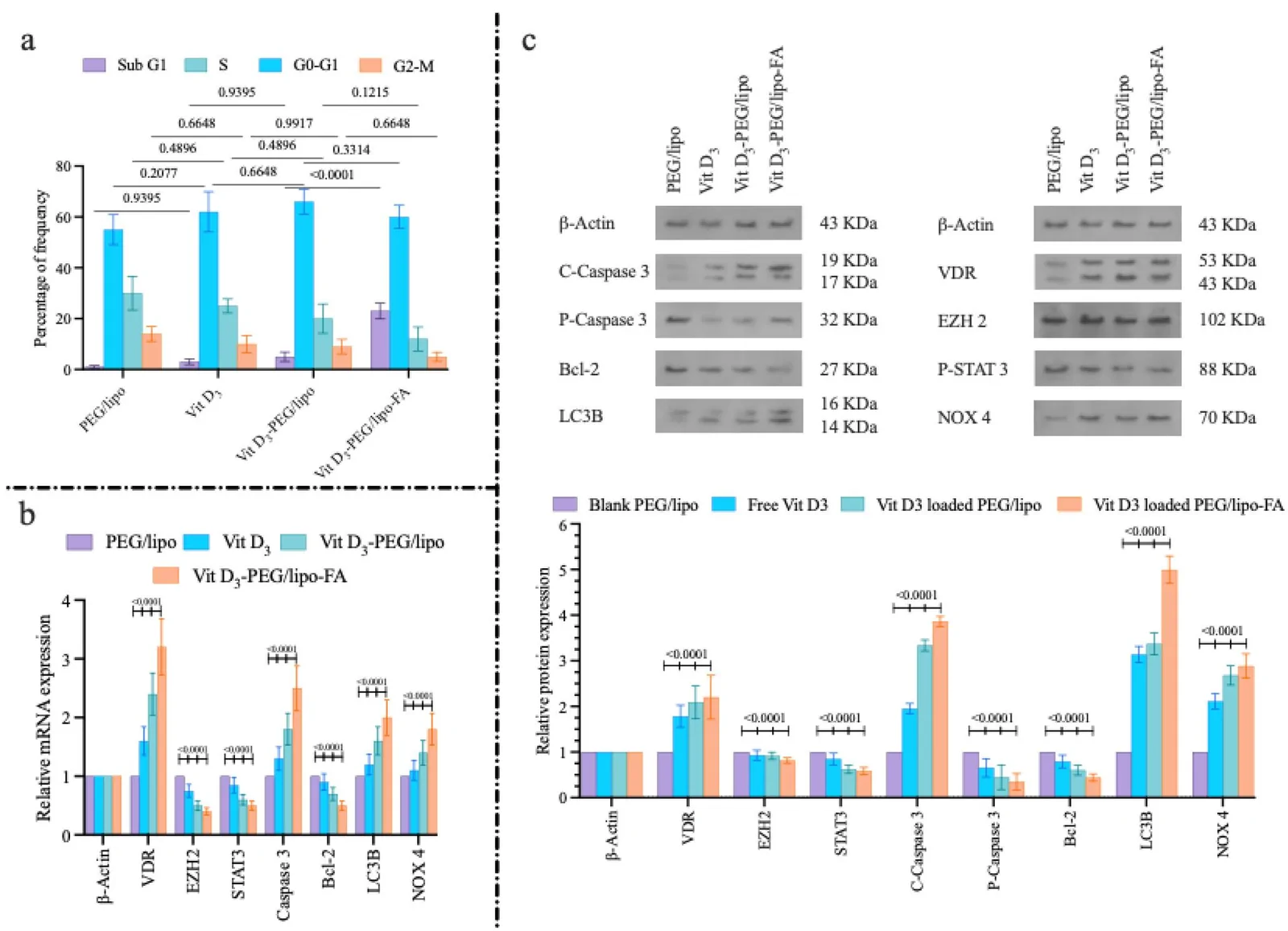
Cell-cycle analysis and molecular responses of MCF-7 cells following treatment with Vit D_3_ formulations. (a) Cell-cycle distribution measured by flow cytometry after 48 h treatment. (b) Relative mRNA expression of VDR, EZH2, STAT3, CASP3, BCL2, LC3B, and NOX4 determined by RT-qPCR. (c) Relative protein expression of the same markers was determined by Western blotting. Data are mean ± SD (n = 3); statistical comparisons are shown in the figure

 Treatment with Vit D_3_-PEG/lipo-FA caused a marked accumulation of cells in the Sub-G1 phase, indicating apoptotic DNA fragmentation (Figure 8a). The Sub-G1 fraction increased from 1.0 ± 0.54% in the PEG/lipo group to 23.0 ± 3.12% in the Vit D_3_-PEG/lipo-FA group (*P* < 0.0001). In parallel, the S-phase population decreased significantly, from 30.0±6.64% to 12.0±4.67% (*P* < 0.0001), while the G2/M fraction showed a non-significant downward trend, from 14.0 ± 2.87% to 5.0 ± 1.76% (*P* = 0.0670). No significant difference was observed in the G0/G1 fraction between the Vit D_3_-PEG/lipo-FA and PEG/lipo groups (60.0 ± 4.58% vs. 55.0 ± 5.87%; *P* = 0.4896). These findings indicate that folate-targeted liposomal Vit D_3_ induces cell-cycle arrest-associated cytotoxicity and promotes apoptotic cell death in MCF-7 cells.

 At the molecular level, RT-qPCR showed that Vit D_3_-PEG/lipo-FA significantly increased VDR expression by 3.2 ± 0.48-fold compared with PEG/lipo controls (*P* = 0.0001). At the same time, EZH2 and STAT3 were significantly downregulated to 0.40 ± 0.06 (*P* = 0.0001) and 0.50 ± 0.08 (*P* = 0.0001), respectively (Figure 8b). In addition, Caspase-3 was upregulated to 2.5 ± 0.38-fold (*P* = 0.0118), whereas Bcl-2 was reduced to 0.50 ± 0.08 (*P* = 0.0001). The autophagy marker LC3B increased to 2.0 ± 0.30 (*P* = 0.0001), and NOX4 increased to 1.8 ± 0.27 (*P* = 0.0001). Western blot analysis confirmed these changes at the protein level (Figure 8c). Vit D_3_-PEG/lipo-FA increased VDR expression and reduced EZH2 and STAT3 protein levels, while also elevating cleaved Caspase-3, LC3B, and NOX4 and decreasing Bcl-2. Together, these data show that folate-targeted liposomal Vit D_3_ reprograms the VDR/EZH2/STAT3 axis and promotes apoptosis, autophagy, ROS generation, and cell-cycle disruption in MCF-7 breast cancer cells.

## Discussion

 This study demonstrates that Vit D_3_–PEG/lipo-FA produces a multi-modal antitumor response in MCF-7 breast cancer cells through improved physicochemical properties, receptor-mediated uptake, pH-responsive release, and coordinated modulation of apoptosis, oxidative stress and autophagy pathways. The combined set of in vitro data supports a mechanism in which targeted delivery both increases intracellular drug delivery and biases cell fate toward programmed cell death rather than uncontrolled necrosis.

 From a formulation perspective, the liposomes exhibit attributes desirable for tumor-directed delivery. The number-weighted mean diameters (≈61 nm for non-targeted drug-loaded liposomes and ≈84 nm for FA-targeted liposomes) fall within the size range favorable for enhanced tumor accumulation via the enhanced permeability and retention (EPR) effect while remaining small enough for efficient cellular uptake.^3^ The moderate negative zeta potential, combined with steric stabilization from PEGylation, is consistent with the observed physical stability over 60 days. In addition, both drug-loaded formulations exhibited very high encapsulation efficiency (>97%) and appreciable drug loading (~9.7–9.8%).^30^ Refrigerated storage for 60 days produced minimal changes in hydrodynamic size and PDI and only modest cumulative leakage (< 9% at day 60), indicating acceptable short-term stability. Hemocompatibility testing also returned reassuring results: negligible hemolysis even at high particle concentrations suggests a low risk of direct erythrocyte damage in systemic exposure scenarios.^31^ Collectively, these physicochemical properties provide a sound delivery vehicle for further biological interrogation.

 Biologically, FA decoration markedly improved functional delivery in folate receptor overexpressing MCF-7 cells: rhodamine-labeled FA-liposomes were internalized more rapidly and to a greater extent than non-targeted liposomes, and this enhanced uptake translated into increased cytotoxic potency (IC₅₀ reduced from ~15 IU·mL⁻¹ for free drug to ~7.2 IU·mL⁻¹ for Vit D_3_–PEG/lipo-FA).^22^ The pH-sensitive release profile similar release at neutral and acidic pH during the first hour followed by significantly greater release at pH 5.5 further supports a tumor- or endosome-selective release mechanism, where acidic compartments amplify payload liberation after receptor-mediated endocytosis.^20^

 At the cellular response level, targeted liposomal Vit D₃ shifted the mode of cell death toward apoptosis and away from necrosis. Encapsulation alone reduced necrosis dramatically (from ~15% to ~3%), and FA targeting increased early apoptotic fractions while maintaining elevated late apoptosis levels an outcome that is typically more favorable therapeutically because it limits pro-inflammatory necrotic responses and promotes controlled cell clearance.^32-34^ The strong increase in the Sub-G1 population and the pronounced inhibition of clonogenic growth (≈90% reduction with FA targeting vs non-targeted liposomes) together indicate both acute cytotoxicity and durable suppression of proliferative potential.^25^

 Molecular profiling was consistent with these phenotypic effects. Vit D_3_–PEG/lipo-FA increased VDR expression while suppressing EZH2 and STAT3, suggesting reprogramming of a pro-survival signaling network. In parallel, Caspase-3 and LC3B were upregulated, Bcl-2 was downregulated, and NOX4 expression and DCFH-DA fluorescence increased, indicating activation of apoptotic signaling, autophagy, and oxidative stress. Importantly, autophagy in cancer can be context dependent: it may serve as a cytoprotective adaptation that helps stressed cells survive, or it may contribute directly to cell death when excessively activated or dysregulated. In the present study, the concomitant rise in LC3B, MDC-positive puncta, apoptosis, and loss of clonogenic survival suggests that autophagy is associated with the overall cytotoxic response, although its exact functional role here remains to be clarified.^35-39^

## Conclusion

 Folate-targeted PEGylated liposomal delivery of vit D_3_ exhibited favorable physicochemical properties sub-100 nm size range, high encapsulation efficiency, and short-term colloidal stability combined with excellent hemocompatibility. In folate-receptor–positive MCF-7 cells, FA decoration markedly enhanced uptake and produced pH-responsive payload release, leading to substantially increased cytotoxicity versus free Vit D_3_ and non-targeted liposomes. Mechanistic findings show coordinated modulation of the VDR/EZH2/STAT3 axis, increased ROS generation, caspase-associated apoptosis, and induction of autophagic markers, together yielding both immediate apoptotic responses and durable suppression of clonogenic growth. These data support a model in which targeted liposomal Vit D_3_ redirects pro-survival signaling toward oxidative-stress–driven apoptosis with concurrent autophagy activation. Overall, Vit D_3_–PEG/lipo-FA is a promising delivery platform to potentiate the antitumor activity of Vit D_3_ and warrants protein-level validation, mechanistic perturbation studies, and in vivo efficacy, pharmacokinetic, and safety evaluations to advance toward clinical translation.

## Study Limitation

 This study has several limitations. First, the findings were obtained in a single breast cancer cell line (MCF-7), which limits generalizability across breast cancer subtypes; therefore, validation in triple-negative and HER2-positive models, as well as in non-cancerous breast cells, is needed to better define tumor selectivity and safety. Second, although key molecular changes were confirmed by RT-qPCR and Western blotting, the causal roles of ROS and autophagy in Vit D_3_-induced cell death were not directly tested using pharmacological inhibitors or genetic knockdown approaches. Third, the work was restricted to an in vitro setting, and therefore, the in vivo efficacy, biodistribution, pharmacokinetics, systemic toxicity, and antitumor activity of the formulation remain unknown. Fourth, stability and hemocompatibility were evaluated only under limited laboratory conditions and over a 60-day refrigerated period; long-term stability and behavior under physiological stress conditions were not assessed. Finally, scale-up feasibility and translational performance have not yet been examined. These issues should be addressed in future preclinical studies.

## Competing Interests

 The authors of the manuscript entitled “pH-Sensitive Folate-Targeted Liposomal Vitamin D_3_ Reprograms EZH2/STAT3 Axis and Induces Apoptosis, ROS, and Autophagy in MCF-7 Breast Cancer Cells” declare no conflicts of interest about the submitted manuscript.

## Ethical Approval

 In reporting our findings, we adhere to the highest standards of publication ethics. This includes transparent disclosure of any potential conflicts of interest and proper acknowledgment of prior work and collaborative contributions. Our primary concern is the safety and welfare of researchers, study participants, and the environment. To ensure this, all laboratory personnel receive thorough training in safe laboratory practices. Strict safety measures are followed when working with cell cultures, hazardous materials, and laboratory equipment. The Tabriz University of Medical Sciences Ethics Committee approved this study (Approval number: IR.TBZMED.VCR.REC.1397.479).
